# Erector Spinae Plane Block for Perioperative Analgesia in a Rabbit

**DOI:** 10.3390/vetsci12100984

**Published:** 2025-10-13

**Authors:** Silvia Scialanca, Giulia Bersanetti, Salvatore Parrillo, Andrea Paolini

**Affiliations:** 1 Small Animal Surgery and Anaesthesia Service, Department of Veterinary Medicine, University of Teramo, 64100 Teramo, Italy; silviascialanca@gmail.com (S.S.); sparrillo@unite.it (S.P.); 2 Clinic for Exotic Animals, Centro Veterinario Specialistico (CVS), Gruppo Animalia Italia, 00137 Rome, Italy

**Keywords:** locoregional anesthesia, ESP block, NAC pain management, rabbit analgesia

## Abstract

**Simple Summary:**

Over the past two decades, anesthetic techniques for nociceptive management in small animal practice have evolved to closely mirror those used in human medicine. It is now widely recognized that effective pain management enhances animal welfare and leads to improved clinical outcomes. Pain management in rabbits is a topic of growing interest in veterinary medicine. The implementation of systemic and local multimodal analgesic protocols is now considered integral to the clinical management of lagomorphs. Nevertheless, the use of locoregional anesthesia techniques in rabbits remains limited and is underrepresented in the current veterinary literature.

**Abstract:**

This clinical case report explores the use of an erector spinae plane (ESP) block to provide perioperative analgesia in a rabbit undergoing spinal decompression surgery. A 6-year-old, 2 kg, spayed, female mixed-breed rabbit presented with acute-onset paraplegia secondary to intervertebral disc extrusion and compressive myelopathy at the L2–L3 disc space. Following neurologic examination and diagnostic evaluation, the patient underwent decompressive surgery at the L2–L3 level. An ultrasound-guided ESP block was performed at the L3 level, with 0.4 mL/kg of ropivacaine 0.5% administered bilaterally. The technique successfully provided intraoperative analgesia and maintained stable hemodynamics without complications. Postoperatively, the rabbit showed a smooth recovery with no need for opioid analgesia. The use of the ESP block was effective in reducing perioperative pain and opioid requirements, highlighting its potential role in multimodal analgesia in rabbits. Further studies are warranted to confirm the safety and efficacy of ESP blocks in this species.

## 1. Introduction

The advantages of locoregional anesthesia techniques are well established in both human and veterinary medicine, including a significant reduction in the use of systemic drugs (particularly opioids), improved cardiorespiratory stability, fewer side effects, and a shorter recovery time [1,2]. Locoregional anesthesia is usually administered as part of a multimodal approach within a varied anesthetic protocol. This approach involves administering a local anesthetic (LA) to a specific area to desensitize the nerve branches responsible for transmitting nociceptive (painful) stimuli. Local anesthetics block transmembrane neuronal sodium channels, thereby interrupting nociceptive transmission to the spinal cord [2,3]. Inter-fascial blocks are a novel locoregional anesthesia technique that has recently been introduced into ultrasound-guided (US) anesthetic practice in dogs and cats [4,5,6,7,8]. In the field of new companion animals (NACs), the available scientific literature is still limited, although it is gradually expanding [9,10,11,12]. These techniques involve the release of an LA within a fascial plane containing the nerves to be desensitized. The erector spinae plane block (ESP) is a US-guided inter-fascial block that provides intraoperative analgesia in both human and veterinary medicine. The ESP block involves the injection of local anesthetic dorsal to the transverse process (TP) and ventral to the erector spinae muscle [13]. The epaxial muscle group includes longissimus dorsi muscles, iliocostalis muscles, and the medial transvers spinalis system [14]. The muscles are located on the dorsal surface of the vertebrae and ribs [14]. The thoracic and lumbar ESP block has been described in canine, feline, and equine cadaveric studies and has consistently resulted in staining of the dorsal branches of the spinal nerves [14,15,16]. Rabbits are among the most popular NACs. There is growing interest in the clinical aspects and anesthetic management of these pets. In particular, the management of nociception and pain in rabbits is a timely and relevant topic, encompassing both systemic analgesia and the use of local anesthetics [17,18,19]. So far, only abdominal inter-fascial blocks [Transversus Abdominis Plane (TAP) and Quadratus Lumborum (QL) blocks] have been described in rabbits [11,20,21,22], and among these, only the clinical case reported by Vettorato et al. was performed in vivo. To the authors’ knowledge, no cadaveric or clinical studies have been conducted on rabbits to investigate the feasibility and efficacy of an ESP block for perioperative analgesia. The following clinical case outlines the technique and perioperative analgesic effectiveness of an ESP block performed at the L3 level in a rabbit undergoing surgery for spinal decompression.

## 2. Case Presentation

A six-year-old, 2 kg, spayed, mixed-breed female rabbit was admitted to the Exotic and Small Mammal Department of the Specialist Veterinary Center (CVS) for acute onset of hindlimb paralysis, anorexia, and signs of discomfort consistent with pain. Clinical and neurological examinations revealed paraplegia with preserved deep pain sensation. Palpation of the cranial lumbar region elicited pain, while spinal reflexes were normal. Based on these findings, a T3–L3 myelopathy was suspected. Blood samples were collected for a complete blood count, biochemistry, and blood gas analysis.

### Anesthetic Management

**Figure 1:**
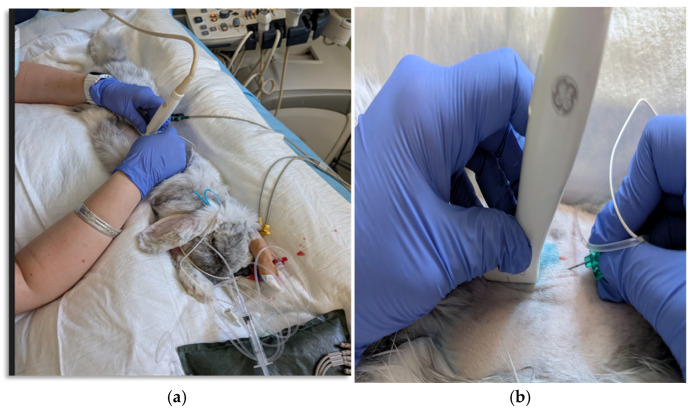
(**a**,**b**) Ultrasound-guided spinal erector block at L4 in rabbits undergoing surgery.

The rabbit was premedicated via an intramuscular (IM) brachial muscle injection of dexmedetomidine 0.07 mg kg^−1^ (0.005 mg mL, Dextroquillan; Fatro, Bologna, Italy), ketamine 5 mg kg^−1^ (100 mg ml, Ketavet; MSD Animal Health, Kenilworth, NJ, USA), and methadone 0.2 mg kg^−1^ (10 mg mL, Semfortan; Eurovet Animal Health, Bladel, The Netherlands). To ensure proper sedation, the rabbit was placed in a dark, stimulus-free room inside a kennel. After 10 min, the level of sedation in the rabbit was sufficient to allow the aseptic placement of two 22-gauge catheters (Jelco; Smiths Medical, Minneapolis, MN, USA) in both cephalic veins. Oxygen was supplemented via a flow by mask at 3 L min^−1^. The trachea was intubated with a 3.5 mm (inner diameter) uncuffed endotracheal tube (Rusch; The Sheridan, Morrisville, NC, USA) using a rigid endoscope and connected to a pediatric Mapleson E breathing system (Intersurgical Ltd., Berkshire, UK) in anesthesia pre-OR. A re-breathing system was used in the OR, and anesthesia was maintained with isoflurane (Isoflo; Zoetis, Rome, Italy) in a 50% mixture of oxygen and air. Advanced imaging using computed tomography (CT) was performed to further investigate the neurological findings. At the level of the L2–L3 intervertebral space, calcified disc material was identified within the vertebral canal in a right paramedian position, causing moderate-to-severe compression of the adjacent spinal cord. Decompressive surgery via L2–L3 right-side hemilaminectomy was therefore considered necessary. An arterial 24-gauge catheter was inserted at the level of the auricular artery for invasive pressure (IBP) monitoring. Heart rate (HR), electrocardiogram (ECG), end-tidal carbon dioxide (CO_2_), end-tidal sevoflurane (ET-Sevo), pulse oximetry (Sp0_2_), and esophageal temperature (T) were constantly monitored before ESP block execution. The patient was positioned in sternal recumbency, and the fur was clipped from T4 to L7, as well as from one side of the dorsal aspect of the transverse processes to the other. Surgical antisepsis was performed. A 5 cm linear array transducer (LA523; Esaote, Milan, Italy) with a frequency of 7–12 MHz was then positioned parallel to the spine at the level of the L3 transverse process. This enabled visualization of the fascial plane located approximately 1 cm deep between the internal intercostal muscles and the erector muscle complex. This plane was defined by the transverse process (Figure 1a,b).

**Figure 2:**
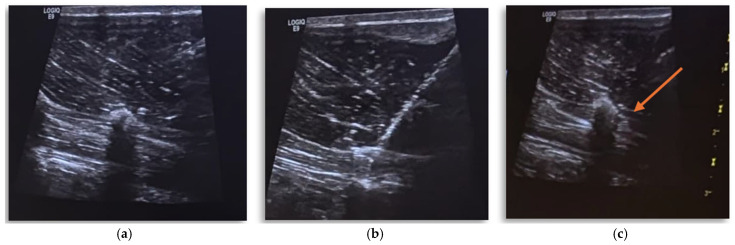
(**a**–**c**) Ultrasound target site of the block. (**a**) Ultrasound window showing the erector spinae complex muscles, transverse process, and vertebral body of L3; (**b**) ultrasound window with insertion of electrostimulation needle at the target site; (**c**) hydro-dissection of the target site in the inter-fascial plane.

The ESP block was performed using a parasagittal, in-plane, US-guided injection of 0.4 mL kg^−1^ of ropivacaine hydrochloride (Ropivacaine Hydrochloride 0.75%, Molteni SpA, Florence, Italy), diluted to 0.5% with sterile NaCl (2 mg kg^−1^ to each side). An electrostimulation needle (B. Braun Stimuplex D SH 22G × 50 mm, Mirandola, Italy) was introduced in plane, in a caudo-cranial orientation, 30 degrees to the ultrasound probe. The needle tip was positioned using ultrasound guidance into the ventral aspect of the erector spine complex muscles and the traverse process of the vertebra L3. To confirm the needle position, 0.2 mL of saline was injected, followed by complete injection of the ropivacaine solution. The same procedure was performed on the other side (Figure 2a–c).

Complications did not occur during the implementation of the blockade. No bradycardia and no hemodynamic changes were observed in the first twenty minutes after administration of ropivacaine. Surgery began 30 min after the ESP block was administered. Intraoperative monitoring included HR; ECG; respiratory rate (*f*R); CO_2_; SpO_2_; T; and systolic, median, and diastolic arterial pressure (SAP, MAP, and DAP) with the IBP system. Response to nociceptive stimulation was defined as an increase in HR, SAP, and/or *f*R > 30% above anesthetic baseline as a measurement of previous time points every five minutes. Fentanyl at 0.005 mg kg^−1^ (0.05 mg’ mL Fentadon; Eurovet Animal Health, Bladel, The Netherlands) was administered as rescue analgesia. During surgery, pressure-controlled intermittent positive-pressure ventilation (IPPV) was applied. A CRI of dexmedetomidine at 0.002 mg kg h^−1^ was administered to reduce the amount of halogenated anesthetic agent required to stabilize the patient and ensure an effective anesthetic plan. The median and range of ETSevo during surgery were 1.1% and [0.9–1.5%]. During spinal decompression, the rabbit’s heart rate dropped below 140 beats per minute (bpm), and its systolic and mean blood pressure dropped to 88 and 56 mmHg, respectively. For this reason, a CRI of dobutamine (Dobutamine chlorydrate; Bioindustria L.I.M. SpA, Novi Ligure, Italy) at 0.005 mg kg^−1^ min^−1^ was administered to correct hypotension and increased on the demand of the rabbit. At the end of the anesthesic procedure, the esophageal temperature was 35.8 °C even though an active warming blanket was provided at the beginning of the surgery. The trachea was extubated 30 min after discontinuing sevoflurane. Recovery was smooth, with a constant-rate infusion of dexmedetomidine at 0.001–0.003 mg kg^−1^ h^−1^ maintained for 24 h post-surgery to guarantee the patient’s immobilization and tranquillization. The rabbit began eating and defecating six hours after being extubated. During the first 24 h postoperatively, meloxicam intramuscular at 1 mg kg^−1^ (5 mg kg Meloxidolor; Le Vet Beheer, Oldeholtpade, The Netherlands) once a day was administered. The Grimace Rabbit Pain Scale was performed one hour after extubation and then every four hours for the first 24 h. From the second day onwards, depending on the scores obtained, pain assessments were spaced out from every 6–8 h to every 12 h. No assessment exceeded the treatment threshold. Postoperative opioids were never administered as rescue analgesia during hospitalization.

## 3. Discussion

The ESP block used during this rabbit’s spinal decompression surgery appeared to be an effective multimodal approach for managing intraoperative nociceptive stimuli and postoperative pain. It is essential for rabbits to resume normal behaviors, such as eating and defecating, after surgery to reduce the risk of complications and ensure a rapid discharge. One of the most complex challenges in companion animals is the assessment of pain in rabbits. Despite improvements in the systemic use of analgesics, recognizing and managing pain continues to be challenging. This is partly due to their characteristic “prayer-like” posture and the lack of multimodal composite pain assessment scales specific to rabbits [18,23]. The assessment of pain stimuli can sometimes be inaccurate due to limited clinical experience or a lack of advanced, validated instruments for recognizing pain, particularly in lagomorphic species [23,24,25]. The implementation of multimodal analgesic and regional anesthetic techniques can decrease the need for systemic analgesics and reduce their potential adverse effects [23]. As aforementioned, inter-fascial blocks are an emerging technique in rabbits. The spread of LA of TAP, QL, and ESP blocks is a controversial issue in the veterinary literature. The injection of the LA in the ESP stains the dorsal branches of the spinal thoracic and/or lumbar nerves with cranial and caudal paravertebral spread [16,26]. The mechanism of action is characterized by neural desensitization and central nervous system inhibition. This is mediated through the diffusion of the local anesthetic into the paravertebral and/or epidural spaces, an effect demonstrated in humans, alongside systemic absorption of the anesthetic [27,28]. Based on evidence from clinical and human cadaveric studies, the most likely primary mechanism involves the direct action of the local anesthetic through its spread and diffusion to neural structures within the fascial plane, located deep in the erector spinae muscles and adjacent tissue compartments. The biological plausibility of this primary mechanism is supported by injectate spread to the ventral rami of the spinal nerves (although this is quite variable) in most studies [27,28,29]. Several veterinary studies have explored various dyeing solutions. Notably, Ferreira et al. [30] demonstrated that, in dogs, the administration of a high volume of LA (0.5 mL/kg) at the level of the transverse process of T5 can result in muscle staining extending from T1 to T9. In the cadaveric study by Portela et al. [14], both high (0.6 mL/kg; HV) and low (0.3 mL/kg; LV) volumes of LA were administered. The results showed that both LV and HV effectively stained the medial and lateral branches across at least four consecutive intervertebral spaces, with a maximum of eight. However, no anesthetic was detected at the level of the ventral branches, epidural space, or paravertebral space. Conversely, Pentosu and colleagues [31] observed a broader spread and greater involvement of the epidural space when performing a retrolaminar thoracolumbar block at the T12 level, using volumes similar to those reported in Portela’s study. While direct comparison between the two studies is challenging due to several variables, it is noteworthy that the extent of epidural involvement in the anesthetic spread remains a topic of ongoing debate for these local-regional techniques. In humans, as mentioned above, peridural involvement has been demonstrated [27]. Other studies in dogs and cats have shown that administering the LA deeper into the aponeurosis of the longissimus lumborum muscle enables visualization of the contrast dye within the ESP compartment [16,26]. The correlation between the mechanism of action and clinical efficacy is not always clear. Firstly, cadaveric studies significantly outnumber clinical studies, despite their inherent limitations, including their inability to accurately replicate living tissue, in which the anesthetic spreads differently. This limitation is also observed in veterinary medicine. Furthermore, the success rate of inter-fascial blocks is highly subjective, depending on factors such as the injection site, volumes, and concentrations used [32].

## 4. Conclusions

In conclusion, the use of an ESP block can be a valid part of a multimodal analgesic protocol in rabbits undergoing hemilaminectomy, thereby reducing the perioperative opioid requirement. Further studies are needed to confirm this finding in rabbit populations. The rabbit did not experience any adverse side effects during the perioperative phase, although further studies are needed to also investigate the effective safety of the block and the potential related complications.

## Data Availability

Data is contained within the article. The original contributions presented in this study are included in the article. Further inquiries can be directed at the corresponding authors.
